# Carbon assimilation and transfer through kelp forests in the NE Atlantic is diminished under a warmer ocean climate

**DOI:** 10.1111/gcb.14303

**Published:** 2018-06-03

**Authors:** Albert Pessarrodona, Pippa J. Moore, Martin D. J. Sayer, Dan A. Smale

**Affiliations:** ^1^ The Citadel Hill Laboratory Marine Biological Association of the United Kingdom Plymouth UK; ^2^ Institute of Biological, Environmental and Rural Sciences Aberystwyth University Aberystwyth UK; ^3^ Centre for Marine Ecosystems Research School of Natural Sciences Edith Cowan University Joondalup WA Australia; ^4^ NERC National Facility for Scientific Diving Scottish Association for Marine Science Oban UK; ^5^ Present address: UWA Oceans Institute and School of Biological Sciences University of Western Australia Crawley WA Australia

**Keywords:** coastal management, *Laminaria hyperborea*, macroalgae, ocean warming, primary productivity, subtidal rocky habitats, temperate reef, trophic subsidies

## Abstract

Global climate change is affecting carbon cycling by driving changes in primary productivity and rates of carbon fixation, release and storage within Earth's vegetated systems. There is, however, limited understanding of how carbon flow between donor and recipient habitats will respond to climatic changes. Macroalgal‐dominated habitats, such as kelp forests, are gaining recognition as important carbon donors within coastal carbon cycles, yet rates of carbon assimilation and transfer through these habitats are poorly resolved. Here, we investigated the likely impacts of ocean warming on coastal carbon cycling by quantifying rates of carbon assimilation and transfer in *Laminaria hyperborea* kelp forests—one of the most extensive coastal vegetated habitat types in the NE Atlantic—along a latitudinal temperature gradient. Kelp forests within warm climatic regimes assimilated, on average, more than three times less carbon and donated less than half the amount of particulate carbon compared to those from cold regimes. These patterns were not related to variability in other environmental parameters. Across their wider geographical distribution, plants exhibited reduced sizes toward their warm‐water equatorward range edge, further suggesting that carbon flow is reduced under warmer climates. Overall, we estimated that *Laminaria hyperborea* forests stored ~11.49 Tg C in living biomass and released particulate carbon at a rate of ~5.71 Tg C year^−1^. This estimated flow of carbon was markedly higher than reported values for most other marine and terrestrial vegetated habitat types in Europe. Together, our observations suggest that continued warming will diminish the amount of carbon that is assimilated and transported through temperate kelp forests in NE Atlantic, with potential consequences for the coastal carbon cycle. Our findings underline the need to consider climate‐driven changes in the capacity of ecosystems to fix and donate carbon when assessing the impacts of climate change on carbon cycling.

## 1 INTRODUCTION

Anthropogenic climate change is disrupting the global carbon cycle, which can further amplify warming through climate‐carbon cycle feedbacks (Friedlingstein, 2015; Raddatz et al., 2007). Climate change can affect the carbon cycling through the biosphere by altering the stocks of carbon held within ecosystems, as well as influencing the efficiency by which it is transferred between different compartments. Although efforts have been made to incorporate climate‐carbon cycle feedbacks into climate projections (Friedlingstein, 2015), they have primarily considered climate‐driven changes in the carbon storage capacity of ecosystems in isolation. For instance, increased tree productivity might increase above‐ground carbon storage in tropical forests (a negative climate carbon feedback; Lewis et al., 2009), while warming might accelerate the release of carbon stored in permafrost soils (a positive carbon feedback; Schuur et al., 2015). Meanwhile, the influence of climate on rates of transfer between compartments of the carbon cycle has been largely overlooked, something that may lead to erroneous predictions of the future carbon sequestration capacity of ecosystems (Sayer, Heard, Grant, Marthews, & Tanner, 2011).

Coastal marine environments exhibit high rates of carbon fixation, export and burial, and in doing so constitute a key component of the global carbon cycle (Bauer et al., 2013). Coastal vegetated habitats (e.g. mangrove forests, seagrass meadows, kelp forests etc.) are some of the most productive ecosystems on Earth and are gaining recognition as important contributors to the oceanic carbon budget (Duarte, Middelburg, & Caraco, 2005; McLeod et al., 2011; Nellemann et al., 2009). A significant fraction of carbon fixed by coastal vegetation flows through detrital pathways, as grazers typically consume a small fraction of total primary productivity (Mann, 1988). Accumulations of detrital material within these ecosystems can form deep organic‐rich soils that represent globally important carbon repositories (Donato et al., 2011; Fourqurean et al., 2012). In addition, the transport of detrital material between habitats represents an important vector of carbon transfer in the coastal marine environment (Hyndes et al., 2014; Smale, Moore, Queiros, Higgs, & Burrows, 2018). Indeed, due to the highly dynamic and open nature of the marine environment, carbon may be buried within depositional habitats great distances from the source, thereby contributing to the total amount of carbon that is buried (Duarte & Krause‐Jensen, 2017).

Macroalgae‐dominated habitats, such as kelp forests, are among the most extensive and productive coastal vegetated habitat types globally (Duarte, Losada, Hendriks, Mazarrasa, & Marbà, 2013), but have been considered to play a secondary role in coastal carbon cycling and storage. This is because (a) macroalgal‐derived matter is assumed to decompose too quickly to allow for long‐range export and burial (Howard et al., 2017); (b) most macroalgae grow on rocks where in situ burial of organic carbon into sediments is precluded (Hill et al., 2015) and; (c) reliable estimates of the amount of carbon fixed and released by macroalgae, as well as their spatial extent, are lacking for most species and regions (Reed & Brzezinski, 2009). A growing body of evidence however, suggests that macroalgae‐derived carbon may be transported to habitats hundreds of kilometers away from source and to depths below thousands of meters (Hobday, 2000). This transfer of carbon constitutes a key trophic subsidy for habitats with low autochthonous productivity, such as offshore sedimentary habitats (Krumhansl & Scheibling, 2012). In addition, macroalgae carbon exports can contribute to carbon storage if they accumulate within habitats with long‐term carbon burial capacity, such as seagrass meadows or offshore depositional sediments (Hill et al., 2015). Furthermore, recent investigations have shown that macroalgal tissues contain refractory carbon compounds (Trevathan‐Tackett et al., 2015), which may represent important organic carbon reservoirs in the ocean (Wada et al., 2008). In light of these recent advances, macroalgal‐dominated habitats are emerging as important donors of carbon within the coastal carbon cycle (Chung, Beardall, Mehta, Sahoo, & Stojkovic, 2011; Hill et al., 2015; Krause‐Jensen & Duarte, 2016). Although climate and other anthropogenic stressors have been shown to alter the carbon stocks contained within marine habitats (Duarte et al., 2013; Fourqurean et al., 2012; Yando et al., 2016), how the flow of carbon between compartments of the coastal carbon cycle will respond to persistent climatic changes remains poorly resolved. Here, we used kelp forests dominated by *Laminaria hyperborea*—which constitute one of the most extensive coastal vegetated habitat types in the NE Atlantic Ocean—as model systems to examine the likely effects of continued ocean warming on carbon stores and fluxes in vegetated coastal ecosystems. Specifically, we quantified (a) the amount of carbon assimilated and stored in living biomass, and (b) the amount of carbon that is donated as particulate detritus through kelp forests persisting under two contrasting thermal regimes.

## 2 MATERIALS AND METHODS

### 2.1 Study sites

**Figure 1:**
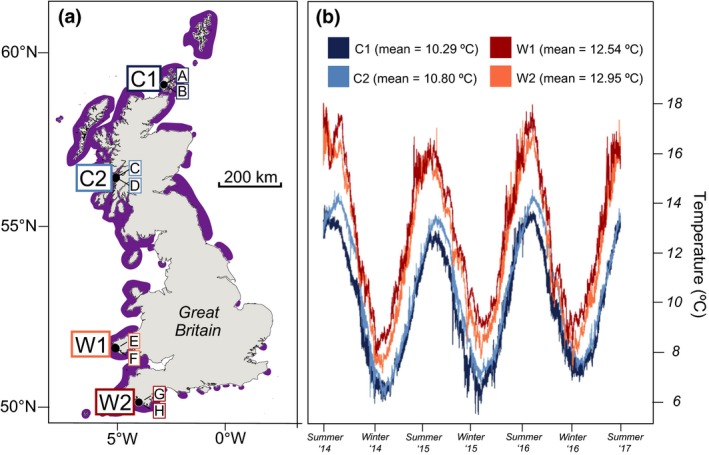
Sampling design and climatic conditions. (a) Positions of the two sampling locations within each climatic regime (C1 = Cold 1, C2 = Cold 2, W1 = Warm 1, W2 = Warm 2), with two sites surveyed within each location (labelled A–H). The approximate distribution of *Laminaria hyperborea* along the coast of Great Britain is also shown with colored shading (b) Temperature profiles (collected with in situ loggers deployed on the reef surface at 3–5 m depth) experienced within each location. Temperature data were collected every 30 min over a 3‐year period [Colour figure can be viewed at http://wileyonlinelibrary.com]

We quantified the amount of organic carbon held within, and donated by, *Laminaria hyperborea* kelp forests at multiple subtidal rocky reef sites situated along a gradient of ~9° of latitude in the NE Atlantic. We sampled two sites within four locations (Figure 1a), which were comparable in terms of key environmental variables (e.g. wave fetch, salinity, nutrients, etc.), but differed with regards to thermal regime (Supporting Information Tables S1, S2 and S3; see also Smale et al., 2016). Sea temperatures within the “cold” locations (hereafter location C1 and C2) were, on average, ~2°C lower compared with the “warm” (W1 and W2) locations (Figure 1b, Supporting Information Table S3); this regional variability in seawater temperature was most evident in summer, when the maximum temperature variability between the coldest and warmest location was 4°C (Figure 1b, Supporting Information Table S3). The surveyed forests extended from the low intertidal to ~10 m depth and were located on wave‐exposed rocky reefs that were similar in terms of geomorphology and topography. Forests were characterized by dense stands of *L. hyperborea*, which was the dominant kelp species (see Smale & Moore, 2017 for details on kelp forest structure).

### 2.2 Carbon assimilation and storage

To characterize the carbon held within kelp forests (i.e. their carbon standing stock), SCUBA divers carried out surveys at ~3–5 m depth (below Chart Datum) in spring (April/May) and summer (August) yearly between 2014 and 2016 at each study site. During each sampling event, the density of *L. hyperborea* was quantified by haphazardly placing eight replicate 1 m^2^ quadrats on hard bedrock and recording the density of mature canopy‐forming plants (plants defined sensu Bolton, 2016). In 2015 and 2016, the carbon standing stock was estimated by multiplying the density of mature *L. hyperborea* plants by their average carbon biomass. At each site, 15 mature plants (i.e. typical canopy‐formers) were randomly sampled by cutting them beneath the holdfast and returning them to the laboratory to measure fresh weight (FW). By sampling kelp plants in different seasons and years, we captured natural variability in kelp standing stock. Sampled plants were spatially dispersed across the site and collected from within the kelp forest (rather than at the canopy edge). To obtain the carbon biomass of each plant, fresh weight (FW) was first converted to dry weight (DW) and then to carbon biomass using an additional conversion factor. To calculate average site‐specific FW:DW ratios, the fresh weight of the 15 complete plants was recorded in 2015, and individual sections of stipe (~10 cm length) and lamina (5 cm strips of both basal and distal material) were removed and dried at ~60°C for at least 48 hr to determine FW:DW ratios for each section. The stipe and basal and distal parts of the lamina were dried separately as the relationship can vary between different parts of the plant (Smale et al., 2016). The FW:DW ratios varied between sites and between parts of the plant (Supporting Information Table S4).

Dry weights were subsequently converted to carbon content using a conversion factor of 0.3125 ± 0.005 (mean ± standard error; Supporting Information Table S5). This factor was a yearly average obtained from routinely sampling two independent kelp populations within the W2 location (50°21′45″N, 4°08′32″W; 50°21′28″N, 4°07′42″W). Sampling was conducted approximately every 2 months to account for seasonal variability in carbon content. During each sampling event, three individual mature *L. hyperborea* plants from each population were collected; kelp tissue from each collected plant was then obtained by sectioning a strip of each kelp lamina along its length (~4 cm width). The samples were freeze‐dried and ground to a fine powder, before quantifying carbon content with a standard elemental analyser (CHN Analyser, EA1110, CE Instruments Ltd, Wigan). The same carbon conversion factor was used to convert all the dry weights from this study (see below).

### 2.3 Carbon donation via particulate detritus

To examine rates of carbon donation to potential receiver habitats, we quantified the release of organic matter as particulate detritus. In *L. hyperborea*, detritus is produced through two main mechanisms: the dislodgment of whole kelp plants from underlying substrata and the loss of lamina tissue. To determine how much detritus is produced via loss of entire kelp plants, we quantified dislodgement rates of mature canopy‐formers. At each study site, three circular plots (~2 m diameter) were established; each plot was ~3 m apart and situated subtidally (again at 3–5 m depth) within the main canopy, rather than on the edge of kelp forest habitat. Within each plot, 15 adult kelps with distinct holdfasts (no fused holdfasts) were tagged by fastening a cable tie sheathed in fluorescent latex surgical tubing around the base of the stipe. Plots were marked with a labeled metal weight and GPS fixed from the surface to aid relocation and to identify specific plots and tagged plants. Dislodgment was quantified over a year; plots were first established in autumn (September 2014) and were revisited the following spring (April 2015; after the winter storms), some 7 months later. Densities of canopy‐forming plants were recorded during each visit using the method previously described**.** The number of remaining tagged plants was recorded and plots were re‐established (using 15 different plants) and then revisited the following autumn, 5 months later (September 2015; total duration of the study 1 year). Tag loss using this method has been observed to be negligible (de Bettignies, Wernberg, Lavery, Vanderklift, & Mohring, 2013) (authors' pers. obs.). To calculate the detrital production resulting from dislodgment of whole plants at each of the sampling periods, we used the following formula as per de Bettignies et al. (2013):

$$\frac{L\times \overset{¯}{D}\times \overset{¯}{w}}{T}$$

Where *T* is the number of tagged kelps (i.e. 15), *L* is the number of lost kelps at each plot, $\overset{¯}{D}$ is the mean *L. hyperborea* density at each site and $\overset{¯}{w}$ is the mean dry weight per plant at each site obtained from our yearly surveys. The results from each plot from the two sampling periods were combined to obtain an annual estimate and then converted to carbon using the conversion factors mentioned above.

In *Laminaria hyperborea*, loss of lamina tissue occurs through two discrete processes: “May cast” and “chronic erosion” (Lüning, 1969; Kain, 1971; Supporting Information Figure S1). May cast is the popular name given to the major detrital pulse arising from the shedding of the previous‐season's lamina growth, which remains attached to newly growing lamina until it is lost entirely as a “growth collar” between March and May (similar to how deciduous trees shed their leaves in autumn). Chronic erosion refers to continuous, gradual erosion of the distal lamina tips that occurs throughout the rest of the year. To estimate the detrital input resulting from the May cast, we randomly selected previous‐season's growth collars attached to canopy‐forming *L. hyperborea* plants and carefully removed and weighed them. At each site, 12–22 growth collars were collected in spring (April–May) 2015 during the shedding period. Fresh weight values were later converted to dry weight and carbon biomass as described above. These values were then standardized per area (g C m^−2^) by multiplying them by the average canopy‐forming plant density at each site obtained from our surveys (2014–2016).

Chronic erosion relates to gradual lamina loss throughout the year and, as it requires more frequent monitoring (i.e. at least monthly surveys), it is far more difficult to quantify across large spatial gradients. Logistical constraints prevented us from conducting monthly sampling at our eight sites simultaneously. To address this issue, we quantified lamina loss rates (through both May cast and chronic erosion) and the relative contribution of each mechanism at two independent, regularly sampled populations; this information was then used to model the annual chronic erosion rates along the latitudinal gradient, based on May cast measurements obtained at the principal study sites. The two independent study populations were located within W2 (50°21′45″N, 4°08′32″W; 50°21′28″N, 4°07′42″W), where *Laminaria hyperborea* dominates the local kelp assemblage. The studied populations extend from the low intertidal into the shallow subtidal zone. We calculated lamina tissue loss monthly from March 2016 to February 2017 using a modified hole‐punch method after Krumhansl and Scheibling (2011). This technique involves punching a series of holes at set distances from the stipe/lamina transition zone, where the primary meristem occurs, to capture growth and loss rates of lamina tissue, and then using biomass‐per‐unit‐of‐length relationships to estimate biomass loss. At each study site, 10 mature canopy‐forming plants were tagged and uniquely labeled every month during spring low tides. A total of three holes were punched in every individual: two at 10 cm and 15 cm above the stipe/lamina transition zone on the central digit, and another one at 15 cm above the aforementioned zone on an outer digit. The two holes punched on the central digit captured the maximal growth in length, which occurs between 2.5 and 15 cm from the central transition zone depending on the month (Kain, 1976), while the hole on the outer digit attempted to capture variability in growth between different digits. The initial length (*L*_i_) of each punched digit was also measured. After a month, tagged kelps were collected by cutting the stipe immediately above the holdfast and returned to the laboratory for analysis.

Final digit lengths (*L*_f_) and final hole positions (*H*_f_), were then recorded for each plant. The mean lamina loss (M; cm) for each plant was obtained by averaging the tissue loss from the central and outer digits (denoted by a subscripted 1 and 2, respectively). Digit loss was obtained by subtracting the final length (*L*_f_) from each digit from the sum of the initial length (*L*_i_) and their respective growth (*G*, cm) as follows:

$$M=\frac{\left[\left(L_{1i}+G_{1}\right)-L_{1f}\right]+\left[\left(L_{2i}+G_{2}\right)-L_{2f}\right]}{2}$$

The growth of each digit was in turn calculated as

$$G_{1}=\left(H_{f1}-10\right)+\left(H_{f2}-15\right)\;and\;G_{2}=\left(H_{f3}-15\right)$$

where *H*_f_ denotes the final position of the holes punched at the central (holes 1 and 2) and outer (hole 3) digits. To convert the loss of distal tissue (cm) to biomass (g), three 5 cm segments from the most distal part of each retrieved lamina were cut, and then weighed (FW). We determined the relationship between fresh and dry weight by drying the outermost segment at 60°C for 48 hr. All FW:DW relationships were highly significant and had an *R* ^2^ ≥ 0.89 (Supporting Information Table S6). We then estimated the dry weight of the rest of the 5 cm segments for each plant using the stated relationship. Finally, the measured and estimated dry biomass per unit length was averaged between all three segments to give the dry biomass per unit length (g/cm) of the distal part of the lamina (*B*_distal_). The daily erosion rate (*E*, g d^−1^ plant^−1^) of lamina tissue from each plant was calculated as:

$$E=M\times B_{\mathrm{distal}}/t$$

Where *M* is the mean lamina loss and *t* denotes the days between the initial and final measurements. The monthly erosion rates were then determined by multiplying the mean daily erosion rates by the total days within a given month. Finally, the relative contribution of the May cast to the total annual production was estimated by dividing the mean detrital production recorded in March and April by the total annual loss of lamina tissue.

Applying our observations of lamina loss from the independent year‐long study, we estimated chronic erosion at each of the sampling sites along the gradient using Monte Carlo simulations. For each sampling site, 1,000 values of May cast production (g DW m^−2^) were generated by sampling randomly from a normal distribution with the obtained mean and standard deviation. Each May cast production value was then randomly assigned to a percentage contribution to the total detritus production (1,000 randomly generated percentages, *p*), which ranged from 56% to 70%, as per our observations from the two independent populations. These values agree with the observations of Lüning (1969), who observed that May cast usually surpasses 50% of the total lamina loss. Chronic lamina erosion (g DW m^−2^) at each site was then calculated as follows:

$$\text{Erosion}=\frac{\left(1-p\right)}{p}\times MC$$

where *p* is the randomly generated percentage contribution and *MC* denotes the randomly generated May cast production obtained from randomly sampling the normal distribution. We then retrieved the mean, standard deviation and standard error from the 1,000 erosion estimates.

Annual estimates of carbon transfer via each mechanism of detritus production (i.e. dislodgement, May cast and chronic erosion; g C m^−2^ year^−1^) were summed for each site to obtain an estimate of total annual carbon flux. We used simple linear regression to examine the relationships between mean sea temperature and site‐level values of carbon standing stock and carbon transfer via particulate detritus production.

### 2.4 Carbon storage and donation across the geographical extent of *Laminaria hyperborea* forests

To assess whether the patterns observed across our study were representative of the wider geographical extent of *Laminaria hyperborea* forests, we compiled kelp biometrics data from study locations across a gradient of 28° of latitude. We used the largest average stipe length recorded for a given age class as a proxy for the maximum biomass (and carbon) accumulation attainable within a given location, as carbon assimilation and storage rates have not yet been measured across the geographical range of *L. hyperborea*. While stipe length is not a direct measure of biomass or carbon assimilation, it is a robust proxy for biomass accumulation for this species, given that: (a) there is a positive relationship between stipe length and plant biomass production (Kain, 1963), (b) stipes are perennial and long‐lived, reaching a maximum length at ~6 years of age (Kain, 1963); (c) mature stipes exhibit minimal seasonal or annual variability in length or biomass (Sjøtun & Fredriksen, 1995); and (d) while stipe length is influenced by a range of factors such as wave exposure and competition for light at local scales (Smale et al., 2016), maximum attainable length is strongly influenced by environmental conditions at regional scales, of which light and temperature are critically important (Rinde & Sjøtun, 2005). As we compared populations only on open coastlines and at similar depths across a broad latitudinal gradient (Supporting Information Table S7), temperature was likely to be a principal driver of variability in maximum attainable stipe length. A similar approach has been used for trees in terrestrial systems (Marks, Muller‐Landau, & Tilman, 2016). Stipe length values were mostly obtained from peer‐reviewed journal publications and published reports, with the exception of unpublished data from Spain (Franco, Tuya and Wernberg unpublished data). Where populations were explicitly stated to be primarily controlled by non‐climatic factors (e.g. sea urchin grazing, light availability), data were excluded from the synthesis. If two or more kelp populations were sampled within a given locality, the highest value was chosen. For references and study details see Supporting Information Table S7.

To provide a first‐order estimate of the overall contribution of *L. hyperborea* forests to coastal carbon cycling, we upscaled the average rates of carbon stock and transfer of kelps from across our study to the approximate global extent of this species, which is endemic to the NE Atlantic region. To the best of our knowledge, there were no other published rates of total particulate carbon release (i.e. all mechanisms of detritus production) for this species that could also be incorporated. Data on the spatial extent of *L. hyperborea* forests were obtained from published papers, government reports and unpublished surveys conducted at a regional‐to‐national scale across Europe (Supporting Information Table S8).

Finally, to contextualize the values of carbon standing stock and carbon donation obtained in this study, we compared *L. hyperborea* forests to other dominant vegetation types in Europe. Coastal vegetation included seagrass (*Posidonia oceanica*) and tidal marshes (*Elytrigia atherica* syn. *Elymus athericus*), while terrestrial vegetation included Norway spruce (*Picea abies*), Scots pine (*Pinus sylvestris*), beech (*Fagus sylvatica*) and temperate and Mediterranean oak (*Quercus robur* and *Q. ilex*). The total carbon standing stock of these systems included the carbon stored in living biomass (above and below ground, i.e. including root systems) and stored in the soils. The particulate detrital carbon flow included all types of litterfall and detritus (e.g. leaves, branches and twigs, reproductive structures). Studies, reviews and meta‐analysis containing data from several sites along latitudinal gradients (as in this study) were preferred. When such studies were not available, those containing the maximum number of different sites were selected. For references and study descriptions see Supporting Information Tables S9 and S10.

## 3 RESULTS

### 3.1 Carbon assimilation and storage

**Figure 2:**
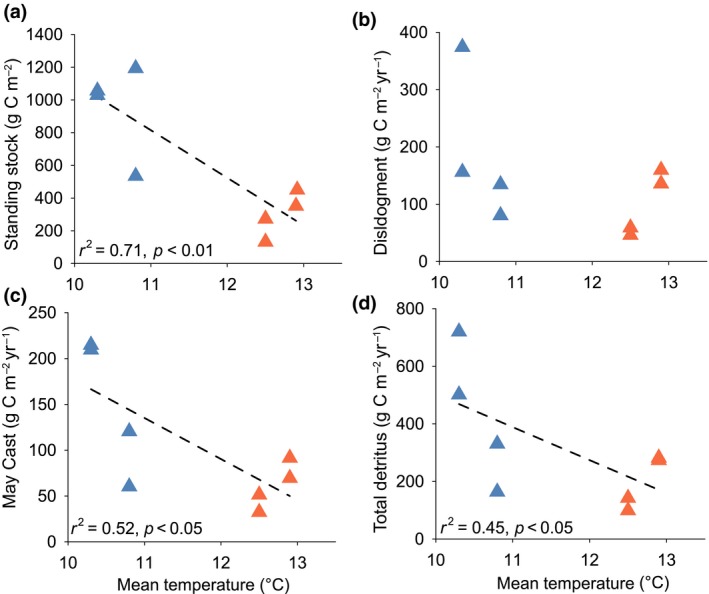
Relationships between mean temperature and mean carbon assimilation and carbon donation via particulate detritus across the eight study sites. Plots indicate the relationship between temperature and carbon standing stock (a), annual carbon release via whole plant dislodgment (b), May cast lamina loss (c), and total annual donation of carbon as detritus (d; i.e. sum of dislodgment, May cast and chronic erosion). Sites within the cold locations are shown in blue and those within warm locations in orange. Dotted lines represent significant relationships (*p* < 0.05) [Colour figure can be viewed at http://wileyonlinelibrary.com]

**Figure 3:**
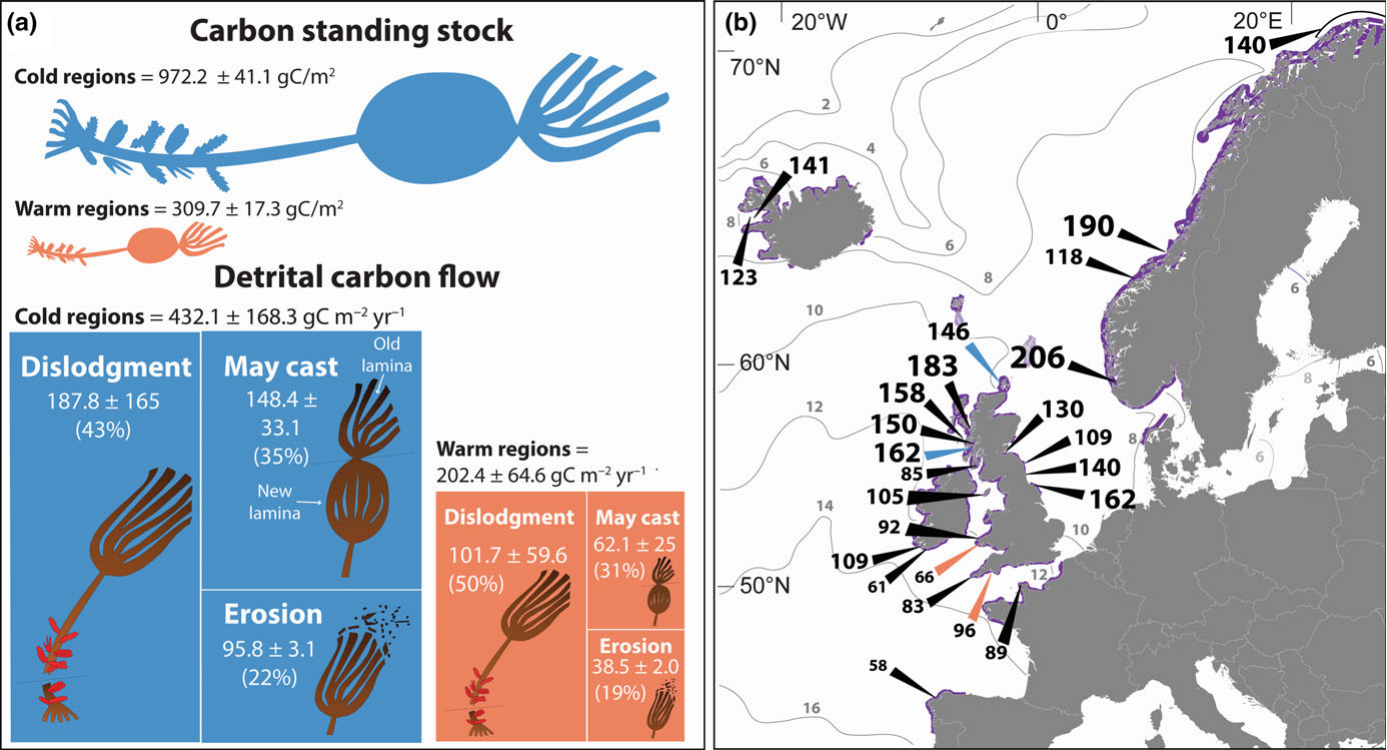
Carbon stock and transfer through *Laminaria hyperborea* forests within our study area and the wider‐scale population structure of *L. hyperborea* in Europe. (a) Standing stock of carbon and annual transfer of carbon as particulate organic matter under cold and warm temperature regimes (means ± SE). Kelp icons representing carbon standing stock are scaled to represent mean values for cold (blue) and warm (orange) locations. Kelp icons within the boxes illustrate the different mechanisms of detrital production: dislodgment of whole plants; May cast resulting from the springtime shedding of the previous‐season's growth collar; and chronic erosion, resulting from the gradual loss of distal lamina tissue. The area of the box comprising each mechanism is scaled to represent study‐wide averages for cold (blue) and warm (orange) locations. (b) Mean maximum stipe length for *L. hyperborea* populations distributed across the species' approximate geographical range (purple line) in the NE Atlantic, collated from various sources (see Supporting Information Table S7 for references and study details). The orange (warm) and blue (cold) arrows indicate sites sampled in the present study; font size increases with stipe length [Colour figure can be viewed at http://wileyonlinelibrary.com]

The structure of kelp forests was spatially variable along the latitudinal gradient. The density of canopy‐forming plants varied considerably between study sites, although there were no clear differences in plant density between climatic regimes (Supporting Information Figures S2, S3). However, average plant biomass did vary with latitude, as plants tended to be higher in biomass at sites within cold locations (Supporting Information Figures S2, S3). Across the study, the average standing stock of carbon (a product of both plant density and size) ranged from 137.4 ± 13.3 g C m^−2^ (mean ± standard error, SE) at a warm site (within W1) to 1198.7 ± 72.8 g C m^−2^ at a cold site (within C2). We recorded a significant negative relationship between carbon standing stock and mean temperature, with sites in the warmer locations generally supporting markedly lower carbon values (Figure 2a). On average, populations in the warm climatic regime stored 68% less carbon than in the cold regime (Figure 3a).

### 3.2 Carbon donation via particulate detritus

Dislodgment of entire plants was the principal mechanism of carbon donation by kelp forests, contributing around half of the total detrital production (Figure 3a). Loss rates of kelp plants through dislodgment ranged between 8.8% and 26.6% during the winter period (September–April) and 4.4%–22.2% during the summer period (April–September). However, because of the high biomass of kelp holdfasts and stipes, which were also exported via the dislodgement of whole plants, these relatively modest rates of plant loss translated to relatively high rates of carbon transfer. The magnitude of carbon flux via dislodgement was highly variable between sites and locations (Supporting Information Figure S3). We did not detect a significant relationship between mean temperature and carbon transfer via dislodgment (Figure 2b), although the highest values were recorded at a cold site (within C1) and the lowest values at a warm site (within W1). The May cast was the second most important mechanism of carbon donation, accounting for 30%–33% of the total detrital production (Figure 3a). In May, when the measurements were obtained, the old growth collar constituted ~40%–60% of the total lamina weight across the study sites. The amount of particulate carbon released during the May cast event declined significantly at higher temperatures (Figure 2c; Supporting Information Table S11), despite marked site‐level variability, particularly across the colder northern locations (Supporting Information Figure S3). Our simulated values of detritus generated through chronic erosion of laminae tissue again showed marked variability between sites, although the three mean highest values were all recorded within the cold regime (Supporting Information Table S12). The three mechanisms of detritus production were combined to quantify the total annual flux of carbon via kelp detritus production. Although between‐site variability was high, the greatest estimated values of carbon transfer were recorded at a site within the C1 location and the lowest a site within the W1 location, with a sixfold difference in total detritus production (Supporting Information Figure S3). Overall, the annual amount of carbon donated via particulate detritus was negatively related to temperature (Figure 2d), with the forests in the warmer locations releasing 54% less particulate carbon than those in colder waters, on average (Figure 3a).

### 3.3 Carbon storage and donation across the geographical extent of *Laminaria hyperborea* forests

Across our study region, site‐averaged maximum stipe length was strongly related to total detritus production (Supporting Information Figure S4), providing support for the use of stipe length as a proxy for detritus production over larger spatial scales. Our synthesis of existing morphological data for *L. hyperborea* populations across its wider range indicated that maximum attainable stipe length is reduced in the warmer lower‐latitude portion of this species' geographical distribution (Figure 3b). In general, the lowest values were recorded for populations situated south of 52°N and the highest values were recorded in the cooler waters around Scotland and Norway (Figure 3b). The maximum attainable stipe length recorded towards the range‐centre, in southern Norway, was 3.5 times greater than the maximum length recorded at the warm, trailing range edge of *L. hyperborea*, on the Iberian Peninsula. The majority of examined studies (83%) had measured multiple individuals within a given size class (Supporting Information Table S7). In most instances, maximum stipe lengths were attained in the older cohorts (>6 years old; Supporting Information Table S7).

A compilation of national‐level assessments indicated that *L. hyperborea* forests extend over an area of at least 18,000 km^2^ in the NE Atlantic region (Supporting Information Table S8). Together with our study‐wide average values (which include both “warm” and “cold” water populations) of carbon standing stock and transfer of carbon via detritus (i.e. 638.2 g C m^−2^ and 317.2 g C m^−2^ year^−1^, respectively), these yield a first‐order estimate of about 11.49 Tg C being held in *L. hyperborea* forests' living biomass and a particulate carbon release rate of about 5.71 Tg C year^−1^.

## 4 DISCUSSION

**Figure 4:**
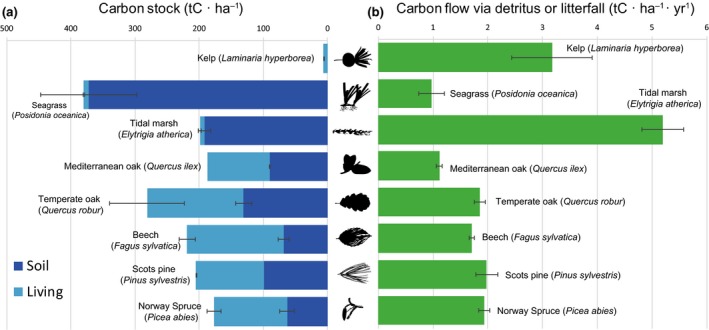
Per area carbon standing stock and carbon flux via detritus for dominant habitat‐forming primary producers in Europe. The carbon stock contained within each habitat is partitioned into the amount stored in soils (dark blue bars) and in living plant tissues (light blue bars), which includes above and below‐ground living biomass. The flow of carbon via detritus includes various kinds of litterfall and leaf shedding and detritus production. Values for *Laminaria hyperborea* are averages across the current study, details and references for other primary producers are provided in Supporting Information Tables S9 and S10. Values are means ± SE [Colour figure can be viewed at http://wileyonlinelibrary.com]

The role of macroalgae in the coastal carbon cycle has recently attracted considerable attention (Chung et al., 2011; Duarte et al., 2013; Hill et al., 2015; Howard et al., 2017; Krause‐Jensen & Duarte, 2016; Reed & Brzezinski, 2009). So far, reliable estimates of macroalgal carbon fluxes in the coastal ocean have been hampered by a scarcity of accurate data of the extent of macroalgal forests and the spatial variability of the amount of carbon they assimilate and release (Reed & Brzezinski, 2009). Using surveys along a large‐scale latitudinal gradient, we have shown that the standing stock of carbon contained within *L. hyperborea* kelp forests is minor when compared with other dominant vegetation types across Europe (Figure 4a). However, the high productivity rates of macroalgae—and kelps in particular—underpin a considerable flux of particulate detrital carbon, which surpasses that of many other vegetated habitats (Figure 4b). It is important to note that our study did not take into account the exudation of dissolved organic carbon, which may represent up to a quarter of the total carbon assimilated and released by *L. hyperborea* (Abdullah & Fredriksen, 2004). Given that *L. hyperborea* forests represent the dominant vegetation type along much of the NE Atlantic coastline, we suggest that changes in the carbon stored and donated by this ecosystem are likely to have important implications for coastal carbon cycling.

Our findings indicate that previous assessments have greatly underestimated the global standing stock of carbon held within kelp forests. For example, based on the best‐available information at the time, Reed and Brzezinski (2009) estimated that the global kelp standing crop was ~7.50 Tg C, which is lower than the ~11.49 Tg C reported here for the range of *L. hyperborea* alone. Given that kelp genera are widely distributed around the world (Teagle, Hawkins, Moore, & Smale, 2017), the total global standing stock could feasibly be an order of magnitude greater than these values. More importantly, by quantifying total detritus production from all mechanisms, we have shown that kelp forests release a considerable amount of particulate organic carbon via detritus (about 5.71 Tg C year^−1^), exceeding per unit area estimates for tree and seagrass species in Europe (Figure 4, right panel). This flow of carbon is likely to be a significant resource subsidy, enhancing the secondary production at receiver habitats (Filbee‐Dexter, Wernberg, Ramirez‐Llodra, Norderhaug, & Pedersen, 2018; Krumhansl & Scheibling, 2012). The transfer of detritus can also alter local species composition and abundance within recipient communities. For example, *L. hyperborea* detritus deposited in fjords has been shown to attract a range of fauna from deep‐sea habitats (Ramirez‐Llodra et al., 2016). The flow of particulate detritus reported here could also be an important source of allochthonous carbon contained within marine carbon sinks such as seagrass meadows or offshore sedimentary habitats (Hill et al., 2015). Even if a minor proportion (e.g. 10%; lower than the average 15% estimated by Krause‐Jensen & Duarte, 2016) of the annual carbon flux estimated here (i.e. ~0.57 Tg C year^−1^) were to reach carbon sink habitats, then *L. hyperborea* forests would make a sizeable contribution to biogenic carbon sequestration, comparable to the 0.72 Tg C ± 0.12 year^−1^ accumulated within European saltmarshes for example (Ouyang & Lee, 2014).

Although the proportion of kelp detritus that reaches carbon sinks is currently unknown, detrital mats comprising *L. hyperborea*‐derived material have been reported at depths in excess of 100s of meters (Freiwald, 1998). In a recent study investigating detritus transport from kelp forests to deep fjords using video cameras, Filbee‐Dexter et al. (2018) recorded the presence of kelp laminae between 400 and 450 m, and estimated its biomass at 22.1 g of fresh weight per m^−2^ of seafloor. In an analysis of benthic sediment cores collected at depths of 70–262 m along the Norwegian coast, Abdullah, Fredriksen, and Christie (2017) suggested that most of the organic matter deposited in the sediments originated from distant *L. hyperborea* forests, as its carbohydrate and phenolic content closely resembled that of the organic fraction. Given their local estimates of organic matter deposition (~0.46 kg C m^−2^ year^−1^) and *L. hyperborea* production (3 kg C m^−2^ year^−1^), they concluded that a substantial proportion of annual kelp production may deposit in the sediments. Still, it is important to note that a dearth of information regarding realized kelp transport pathways, residence times and burial rates of kelp‐derived matter hinders our understanding of the relative contribution of kelp donation to carbon sinks.

Across the latitudinal gradient examined here, populations in the warm locations stored an average of 68% less carbon and released 53% less particulate carbon than those from colder northernmost populations. The markedly lower carbon standing stock and rates of particulate carbon release observed under the warm climate regime was principally the result of the smaller size of *L. hyperborea* plants in those areas (Supporting Information Figure S3). Although multiple environmental variables such as nutrient availability, irradiance and wave exposure influence the size, morphology and productivity of kelp plants (Pedersen, Nejrup, Fredriksen, Christie, & Norderhaug, 2012; Smale et al., 2016), they did not co‐vary with temperature or differ between the climate regimes (Supporting Information Table S2). Biological factors such as grazing pressure are also known to affect kelp standing biomass (Estes & Palmisano, 1974). However, the principal kelp‐grazers in our study area (an omnivorous sea urchin and several species of gastropod mollusc) are not considered to exert strong top‐down control on kelp populations, as they are small and do not form dense grazing aggregations (Hargrave, Foggo, Pessarrodona, & Smale, 2017; Smale et al., 2013). Reduced plant productivity (and therefore lower potential for carbon assimilation and potential storage and release) is frequently observed in temperature‐stressed populations, such as those living at the warm edge of their distribution (Hatcher, Kirkman, & Wood, 1987), or those experiencing frequent heat and drought stress (Allen, Breshears, & McDowell, 2015). Our findings agree with previous historical (John, 1968; Whittock, 1969) and more recent (Smale et al., 2016) latitudinal surveys across the study region, which also found reduced kelp sizes and productivity in southern Great Britain compared to northern sites. We found a similar pattern across the geographical range of *L. hyperborea* when examining maximum stipe size, a reliable proxy for plant productivity and detritus production (Kain, 1971). Although the quantity of morphological data from marginal populations at the trailing range edge is limited, Pereira, Engelen, Pearson, Valero, and Serrão (2017) recently showed that *L. hyperborea* individuals in Portuguese tidal pools rarely surpass 70 cm in total length, while unpublished data from subtidal populations in northern Spain indicate that mean maximum stipe length does not exceed 60 cm at the warm rear‐edge (Franco et al., unpublished; Supporting Information Table S7). In contrast, mature plants from Norway, Iceland or northern Great Britain often surpassed 120 cm in stipe length, with the greatest stipe lengths documented at the central portion of the species range (Supporting Information Table S7). This strongly suggests that biomass accumulation, primary productivity and carbon transfer associated with *L. hyperborea* forests is reduced under warmer conditions.

Together with the available evidence from the literature, our findings indicate that continued ocean warming is likely to diminish the donor capacity of kelp forests in the NE Atlantic Ocean. Sea temperatures in this region have increased significantly in recent decades (Belkin, 2009), with a further 1.5–5°C of warming predicted for this century (Philippart et al., 2011). *L. hyperborea* has already undergone a range contraction of ~250 km at its warm, trailing range edge over the past 40 years (Assis, Lucas, Bárbara, & Serrão, 2016), and further losses are expected as the water continues to warm (Assis et al., 2016; Müller, Laepple, Bartsch, & Wiencke, 2009). The loss of *L. hyperborea* forests at the warmer portions of its range is no exception, as several other Atlantic kelp species have exhibited climate‐related declines in abundance and spatial extent in recent years (Fernández, 2016; Filbee‐Dexter, Feehan, & Scheibling, 2016; Raybaud et al., 2013). Such losses of marginal kelp populations at trailing range edges have likely led to reductions in the magnitude of carbon assimilation and donation through kelp forests in the North Atlantic region.

Warming can extend the growing season and lead to increases in overall vegetation productivity at boreal latitudes (Sturm et al., 2001), which could in principle compensate for reductions in temperate regions. For instance, reductions in sea ice cover in the Arctic are expected to promote growth and facilitate the poleward range expansions of kelp and seagrasses, with consequent increases in benthic primary production and alterations to inshore carbon cycling (Krause‐Jensen & Duarte, 2014). However, reductions in sea ice extent may in fact negatively affect primary productivity and carbon assimilation as they allow for wind‐driven resuspension of sediments (Bonsell & Dunton, 2018). In addition, benthic productivity in the Arctic will still nonetheless be greatly restricted by long periods of darkness (Dunton, 1985). Finally, many cold‐temperate macroalgae species are expected to make only moderate gains in the Arctic (Müller et al., 2009), which may not compensate for the projected losses at the trailing range edge. For instance, despite expected range expansion into the Arctic, *L. hyperborea* is still predicted to lose between 8.41% and 39.44% of its global suitable habitat by the end of the century, depending on the emissions scenario (Assis et al., 2016). Although the extent to which the poleward migration of warm‐water species will offset for the trailing‐edge decline of cold‐affinity species remains unclear, the majority of highly productive kelp species in the NE Atlantic have cold northerly distributions (Müller et al., 2009; Raybaud et al., 2013). Projected increases in CO_2_ levels could, theoretically, have a positive effect on future kelp productivity and carbon assimilation (Brodie et al., 2014), which may compensate for temperature‐meditated declines. In many kelp species however, photosynthesis is already carbon saturated under present conditions, and elevated CO_2_ levels do not lead to increased primary production and growth rates (Iñiguez et al., 2016). In reality, elevated CO_2_ levels may facilitate kelp competitors such as mat (turf)‐forming algae (Connell, Kroeker, Fabricius, Kline, & Russell, 2013), which can displace kelp populations under stressful conditions, such as extreme ocean warming or reduced water quality (Filbee‐Dexter & Wernberg, 2018).

The observed decline of kelp forests in the Atlantic reflects patterns in other temperate regions of the global ocean. Recent warming has been linked with loss of kelp forests and other large canopy‐forming macroalgae in several systems (e.g. Filbee‐Dexter et al., 2016; Johnson et al., 2011; Vergés et al., 2014; Wernberg et al., 2016), leading to declines in detrital production (Krumhansl et al., 2014) and, intuitively, carbon fluxes. In a recent meta‐analysis of kelp forest change over the past half‐century, Krumhansl et al. (2016) reported significant declines in kelp abundances in 38% of the global ecoregions analyzed, while 28% of regions registered increases. Nevertheless, some of the regions where populations were found to be stable, or even increasing, have experienced massive declines and even extirpations of kelp populations in recent years (e.g. the South European Atlantic shelf; Fernández, 2011; Raybaud et al., 2013; Assis et al., 2016, 2017). Continued declines in kelp forest extent, together with declines in productivity and shifts in species composition, could presumably diminish carbon assimilation and transfer through vegetated temperate marine ecosystems globally, with potential consequences for carbon cycling in the coastal ocean. Evidence to‐date suggests that kelp losses may only be partly compensated by moderate range expansion into polar regions, with productivity still being severely restricted by sea ice dynamics (Bonsell & Dunton, 2018).

Until now, most efforts have been focused on understanding how climate change might affect the carbon sequestration and storage capacity within ecosystems. It is becoming evident, however, that climate‐driven alterations in the fluxes between different compartments can also alter processes within the carbon cycle. For instance, climate change is predicted to increase riverine carbon exports (Larsen, Andersen, & Hessen, 2011), a significant component of the global carbon cycle which connects terrestrial and oceanic carbon reservoirs. Our work suggests that such alterations may also be important in the coastal ocean, where populations, habitats and trophic resources are highly interconnected because of the open and dynamic nature of the marine environment. Moreover, our results indicate that the magnitude of carbon transfer via detritus in coastal vegetated habitats is greater than previously reported, highlighting the need to incorporate this process into coastal biogeochemical models. Considering climate‐driven changes in the carbon donor capacity of ecosystems will improve our understanding of how carbon cycle pathways will change in a future warmer ocean.
