# Supplementary material for: Carbon assimilation and transfer through kelp forests in the NE Atlantic is diminished under a warmer ocean climate
Source: Glob Chang Biol. 2018 Jun 3;24(9):4386–98. doi: 10.1111/gcb.14303 (PMC6120504; doi:10.1111/gcb.14303)
Supplement: Supplementary file 1 [file GCB-24-4386-s001.docx]

SUPPORTING INFORMATION

**Carbon assimilation and transfer through kelp forests in the NE Atlantic is diminished under a warmer ocean climate**

Albert Pessarrodona, Pippa J. Moore, Martin D. J. Sayer, Dan A. Smale

**Table S1** Summary of environmental conditions at each study site. This study included 8 sites within 4 distinct locations in the UK. ‘Depth’ indicates average depth (below chart datum) of each study site. ‘Mean SST’ is the annual mean temperature calculated from satellite-derived sea surface temperature (SST) data (2005-2014, 9 km resolution data from Pathfinder AVHRR satellite). ‘Log Chl *a* mean’ is the average annual concentration of chlorophyll *a* for each site (log10 mg m^−3^ from MODIS Aqua satellite data, 2002–2012). ‘Log wave fetch’ is a broad-scale metric of wave exposure, derived by summing fetch values calculated for 32 angular sectors surrounding each study site (Burrows, 2012). ). ‘PO_4_^3-^ʼ and ‘NO_3-_ +NO_2-_ʼ indicate average concentrations of phosphate and nitrite + nitrate respectively (n = 2 water samples taken from ~1 m above the kelp canopy in both spring and summer 2015).

| **Location** | **Site** | **Locality** | **Lat/Long** | **Depth (m)** | **Mean SST (°C)** | **Log Chl *a* mean**  **(mg m^-3^)** | **Log wave fetch (km)** | **PO_4_^3-^ (μM)** | **NO_3_^-^+NO_2_^-^ (μM)** |
| --- | --- | --- | --- | --- | --- | --- | --- | --- | --- |
| C1 | A | Warbeth Bay, N Scotland | 58.9577, -3.3377 | 3 | 9.7 | 0.21 | 3.8 | 0.17 | 1.66 |
| C1 | B | N Graemsay, N Scotland | 58.9407, -3.2937 | 4 | 9.8 | 0.26 | 3.5 | 0.23 | 1.92 |
| C2 | C | Dubh Sgeir, W Scotland | 56.3661, -5.6213 | 5 | 10.8 | 0.59 | 3.3 | 0.30 | 2.93 |
| C2 | D | W Kerrera, W Scotland | 56.3925, -5.5794 | 4 | 10.7 | 0.65 | 3.1 | 0.26 | 2.52 |
| W1 | E | Stack Rock, SW Wales, | 51.7743, -5.1744 | 5 | 11.7 | 0.43 | 3.7 | 0.16 | 3.17 |
| W1 | F | Mill Haven, SW Wales | 51.7681, -5.1683 | 4 | 11.8 | 0.43 | 3.5 | 0.22 | 2.68 |
| W2 | G | Hillsea Pt., SW England | 50.2995, -4.0151 | 3 | 12.4 | 0.28 | 4.1 | 0.17 | 2.41 |
| W2 | H | NW Mewstone, SW England | 50.3080, -4.1083 | 4 | 12.5 | 0.43 | 3.5 | 0.11 | 3.05 |

**Table S2** Welch’s t-tests to examine differences in environmental variables (listed in Table S1) between the ‘cold’ and the ‘warm’ temperature regime.

| **Variable** | **T value** | **P value** |
| --- | --- | --- |
| Depth | 0.00 | 1.000 |
| Mean SST (°C) | 5.21 | **0.003** |
| Log Chl *a* mean  (mg m^-3^) | 0.29 | 0.783 |
| Log wave fetch (km) | 1.34 | 0.230 |
| PO_4_^3-^ (μM) | 1.70 | 0.141 |
| NO_3_^-^+NO_2_^-^ (μM) | 2.14 | 0.081 |

**Table S3** Summary of broad-scale environmental variables for each location. Temperature data were collected *in situ* at one site within each location, for the period July 2014 to July 2018, using a ‘Tidbit’ temperature logger (Onset, USA) attached to the seabed; the mean and maximum values recorded in each location are shown. Maximum tidal range was obtained from the nearest station in the UK Tide Gauge Network to the study sites (Wick in N Scotland, Tobermory in W Scotland, Milford Haven in W Wales and Devonport in SW England). The network was established in 1953 and sea level data are managed and accessed through the British Oceanographic Data Centre ([www.bodc.ac.uk](http://www.bodc.ac.uk)). Regional irradiance levels were inferred from average annual sunshine hours (1981-2010) obtained from the nearest weather station to the study sites (Loch of Hundland in N Scotland, Dunstaffange in W Scotland, Milford Haven in W Wales and Mountbatten in SW England); weather data are collected, managed and made accessible by the UK Met Office ([www.metoffice.gov.uk](http://www.metoffice.gov.uk)). Finally, all study sites were situated outside any influence from freshwater input from rivers and estuaries and were fully marine in nature. See Dye *et al.* (2013) for regional salinity values.

| **Location** | **Locality** | **Mean *in situ* temperature (°C)** | **Max. summer temperature (°C)** | **Max tidal range (m)** | **Annual sunshine (hr yr^-1^)** | **Salinity (p.s.u)** |
| --- | --- | --- | --- | --- | --- | --- |
| C1 | N Scotland | 10.3 | 14.0 | 3.6 | 1197 | ~35 |
| C2 | W Scotland | 10.8 | 14.6 | 4.7 | 1219 | ~35 |
| W1 | SW Wales | 12.5 | 17.9 | 7.3 | 1625 | ~35 |
| W2 | SW England | 12.9 | 18.0 | 5.6 | 1730 | ~35 |

| **Location/Site** | **C1 (A)** | **C1 (B)** | **C2 (C)** | **C2 (D)** | **W1 (E)** | **W1 (F)** | **W2 (G)** | **W2 (H)** |
| --- | --- | --- | --- | --- | --- | --- | --- | --- |
| Lamina base | 0.32 ± 0.07 | 0.30 ± 0.07 | 0.22 ± 0.03 | 0.24 ± 0.03 | 0.31 ± 0.04 | 0.24 ± 0.03 | 0.31 ± 0.05 | 0.29 ± 0.03 |
| Lamina distal | 0.15 ± 0.04 | 0.18 ± 0.11 | 0.14 ± 0.04 | 0.14 ± 0.02 | 0.14 ± 0.05 | 0.15 ± 0.02 | 0.19 ± 0.04 | 0.15 ± 0.02 |
| Stipe | 0.29 ± 0.05 | 0.23 ± 0.07 | 0.23 ± 0.06 | 0.28 ± 0.10 | 0.18 ± 0.03 | 0.16 ± 0.02 | 0.17 ± 0.02 | 0.18 ± 0.03 |

**Table S4** Average fresh weight (FW) to dry weight (DW) ratios (FW:DW) from of the stipe, and basal and distal portions of the lamina at each of our latitudinal study sites (n=16 per site and part). These were obtained by weighing sections of fresh stipe (~10 cm length) and lamina (5 cm strips of both basal and distal material) and reweighing them after drying at ~60°C for at least 48 hours. These values were used to convert fresh biomass measurements to dry biomass.

**Table S5** Monthly and yearly carbon content (g C · g DW; Mean ± Standard Deviation) from two independent, long-term *L. hyperborea* populations located within W2. Three plants from each site were sampled on every event. The yearly total average (i.e. 0.3125, in bold) was used to convert dry biomass to carbon biomass throughout the study.

| **Month** | **Site 1** | **Site 2** | **Total average** |
| --- | --- | --- | --- |
| April | 0.3000 ± 0.0071 | 0.2769 ± 0.0085 | 0.2885 ± 0.0143 |
| June | 0.2837 ± 0.0057 | 0.2762 ± 0.0035 | 0.2799 ± 0.0059 |
| July | 0.3140 ± 0.0106 | 0.3087 ± 0.0143 | 0.3113 ± 0.0123 |
| September | 0.3336 ± 0.0086 | 0.3312 ± 0.0074 | 0.3324 ± 0.0077 |
| November | 0.3538 ± 0.0091 | 0.3386 ± 0.0189 | 0.3462 ± 0.0162 |
| January | 0.2910 ± 0.0245 | 0.3046 ± 0.0223 | 0.2978 ± 0.0234 |
| **Year** | 0.3156 ± 0.0278 | 0.3097 ± 0.0270 | **0.3125 ± 0.0273** |

**Table S6** Monthly FW:DW relationships from the distal portion of the lamina, and its R^2^, from two independent, long-term *L. hyperborea* populations located within W2. To obtain these relationships, 5 cm segments from the most distal part of each retrieved lamina were cut, and then weighed (FW) every month (n=10 per site). The segments were then dried at 60°C for 48 h. These values were then used to estimate the loss of lamina lost due to ‘chronic’ erosion each month.

|  | **Site 1** | | **Site 2** | |
| --- | --- | --- | --- | --- |
| **Month** | Equation | R² | Equation | R² |
| March | y = 0.1954x - 0.3421 | 0.92 | y = 0.245x - 2.567 | 0.96 |
| April | y = 0.1472x + 0.107 | 0.99 | y = 0.1458x + 0.0302 | 0.97 |
| May | y = 0.1375x - 0.096 | 0.99 | y = 0.124x + 0.0467 | 0.95 |
| June | y = 0.1339x + 0.0891 | 0.95 | y = 0.1114x + 0.1465 | 0.93 |
| July | y = 0.1692x - 0.14 | 0.99 | y = 0.1362x + 0.3021 | 0.96 |
| August | y = 0.1713x - 0.2307 | 0.91 | y = 0.23x - 1.494 | 0.98 |
| September | y = 0.1772x - 0.5431 | 0.89 | y = 0.2001x - 0.8453 | 0.97 |
| October | y = 0.1431x + 0.2676 | 0.97 | y = 0.1392x - 0.0572 | 0.97 |
| November | y = 0.2001x - 0.9497 | 0.92 | y = 0.1427x + 0.6226 | 0.90 |
| December | y = 0.1705x - 0.3244 | 0.99 | y = 0.171x - 0.6682 | 0.91 |
| January | y = 0.1763x - 0.3226 | 0.91 | y = 0.1837x - 0.4186 | 0.95 |
| February | y = 0.1533x - 0.1119 | 0.97 | y = 0.168x - 0.4206 | 0.90 |

**Table S7** Mean maximum stipe length of different *Laminaria hyperborea* populations across its endemic range in the northeast Atlantic, as shown in Figure 3(b). The mean maximum stipe length attainable for any given age class is shown. If two or more populations were sampled within a given locality, the highest value was chosen. Values were obtained either from the raw data, the original text or the graphs by using the graph digitizing tool WebPlotDigitizer.

| **Locality** | **Latitude** | **Longitude** | **Country** | **Year** | **Depth (m)** | **Age (year)** | **Max. stipe length (cm)** | **Reference** | **Method** |
| --- | --- | --- | --- | --- | --- | --- | --- | --- | --- |
| Finmark | 70.266/71.196 | 24.185/31.062 | Norway | 1985 | 5 | 18 | 139.55 | (Sjøtun *et al.*, 1993; Rinde & Sjøtun, 2005) | Graph digitizer |
| Langley | 65.394307 | -22.98533 | Iceland | 1977 | 3 | 12 | 141 | (Gunnarsson, 1991) | Graph digitizer |
| Oddbjarnarsker | 65.326748 | -23.09245 | Iceland | 1978 | 3 | 10 | 122.64 | (Gunnarsson, 1991) | Graph digitizer |
| Station 107B, | 63.977916 | 9.762678 | Norway | 1999 | 4-5 | 9 | 190 ± 28 | (Sjøtun *et al.*, 2000) | In text |
| Molde Archipelago | 62.816667 | 6.4 | Norway | 2006 | 5-7 | 7 | 118 ± 11.4 | (Pedersen *et al.*, 2012) | In text |
| Finnøy archipelago | 59.132341 | 5.76431 | Norway | 2008 | 5 | 8 | 206 ± 0 | (Bekkby *et al.*, 2014) | In text |
| Warbeth Bay | 58.9577 | -3.3377 | Britain | 2014-2015 | 3 | 6 | 146.5 ± 12.5 | (Smale *et al.*, 2016) | Raw data |
| Arisaig | 56.949587 | -5.863207 | Britain | 1969-1970 | 3 | 9 | 183 ± 0 | (Jupp & Drew, 1974) | In text |
| Soa Island (East) | 56.56741 | -6.625631 | Britain | 1967-1968 | 4.6 | 7 | 157.6 ± 3 | (John, 1968) | In text |
| Connel Sound | 56.457977 | -5.418204 | Britain | 1976 | 4 | 9 | 150 ± 0 | (Kain, 1977) | In text |
| Dubh Sgeir | 56.3661 | -5.6213 | Britain | 2014-2015 | 5 | 11 | 162.05 ± 7.1 | (Smale *et al.*, 2016) | Raw data |
| Pettico Wick | 55.915161 | -2.150308 | Britain | 1968 | 6 | 8 | 130 ± 22 | (Whittock, 1969) | In text |
| Beadnell-Lady's Hole | 55.553219 | -1.623398 | Britain | 1967-1968 | 3 | 7 | 109 ± 4.2 | (John, 1968) | In text |
| Saddal Bay | 55.455736 | -5.501283 | Britain | 1967-1968 | 3.6 | 7 | 84.6 ± 5.6 | (John, 1968) | In text |
| Marsden - Byer's Hole | 54.977398 | -1.372082 | Britain | 1967-1968 | 2 | 7 | 140 ± 5.6 | (John, 1968) | In text |
| Redcar - Salt Scar | 54.618789 | -1.044643 | Britain | 1967-1968 | 4.2 | 7 | 180 ± 19.9 | (John, 1968) | In text |
| Forest 2, Port Erin | 54.09025 | -4.777385 | Britain | 1959 | 2 | 7 | 105 | (Kain, 1963) | Graph digitizer |
| Cardigan Island | 52.131861 | -4.689214 | Britain | 1967-1968 | 3.6 | 7 | 92 ± 2.8 | (John, 1968) | In text |
| Mill Haven | 51.7681 | -5.1683 | Britain | 2014-2015 | 4 | 7 | 66.08 ± 15.6 | (Smale *et al.*, 2016) | Raw data |
| Dunmanus Bay | 51.58333 | -9.75 | Ireland | 1968 | 5 | 7 | 109 ± 9.6 | (Whittock, 1969) | In text |
| Carrigathorna | 51.489439 | -9.292027 | Ireland | 1972 | 3 | 6 | 61 ± 4.8 | (Norton *et al.*, 1977) | In text |
| NW Mewstone | 50.308 | -4.1083 | Britain | 2014-2015 | 4 | 9 | 96 ± 0 | (Smale *et al.*, 2016) | Raw data |
| Sennen Cove | 50.083333 | -5.416667 | Britain | 1967 | 3 | 7 | 83 ± 0 | (Whittock, 1969) | In text |
| Port Levi | 49.69997 | -1.476134 | France | 1975-1976 | 6 | 9 | 89.86 | (Sheppard *et al.*, 1978) | Graph digitizer |
| A Coruña | [43.365](https://tools.wmflabs.org/geohack/geohack.php?pagename=A_Coru%C3%B1a&params=43.365_N_8.41_W_type:city(244388)_region:ES-C) | -8.410 | Spain | 2013 | 6 | 6-8 | 57.7 ± 4.5 | Franco, Tuya & Wernberg unpubl. data | Raw data |

**Table S8** Available estimates of the areal extent of *Laminaria hyperborea* forests in European countries

| Country | Area (km^2^) | Notes | Reference |
| --- | --- | --- | --- |
| Portugal | ? | Distribution spans from Vila do Conde to the border with Spain, ~70 km of coastline. | (Assis *et al.*, 2016) |
| Spain | 827 | From N and NW Spain. | (Fernández, pers. comm.) |
| France | 1990* | From surveys of *L. hyperborea* forests in Brittany. | (Chassé & Kerambrun, 1988) |
| Denmark & Germany | ? |  |  |
| Britain & Ireland  Ireland  Scotland | 15984  392  2155–5933 | Using habitat suitability models based on the environmental preferences of the species.  The authors estimated that kelp forests are found along 3920 km of Ireland’s coastline. They considered an average width of kelp beds of about 100 m, based on surveys performed in Galway.  Based on statistical models and ecological data. The lesser value refers to areas where kelp is most likely to be abundant, while the greater value corresponds to areas where there is a 25% probability of finding kelp*. L. hyperborea* is the dominant kelp in Scotland. | (Yesson *et al.*, 2014)  (Werner & Kraan, 2004)  (Burrows *et al.*, 2014) |
| Norway | 5904  5000–10000 | Based on statistical models and ecological data. The maximum extent of *L. hyperborea* forest in Norway is estimated at 7883 km^2^, if sea urchin grazing and overgrowing of kelp by filamentous algae did not exist.  Based on research on *L. hyperborea* for commercial harvesting. | (Gundersen *et al.*, 2010)  (Jensen, 1998) |
| Iceland | ? |  |  |
| **Europe total** | ~18000 | Taking a conservative estimate of 9000 km^2^ for Britain and Ireland (given the 15984 km^2^ predicted by Yesson *et al.* 2014), 5904 km^2^ for Norway and considering that the extent of *L. hyperborea* in the areas there are no reliable estimates is ~280 km^2^. |  |

*Does not include other sections of French coastline were *L. hyperborea* is present

**Table S9** Data sources used to quantify the standing stock of carbon associated with dominant European habitat-forming species, as shown in Figure 4. ‘n’ refers to the number of sites, plots or stands sampled in each study. If the estimates for the living biomass and soil carbon stock were obtained from different studies, they are separated by a comma.

| **Common name** | **Species** | **n** | **Study locations and descriptions** | **Reference** |
| --- | --- | --- | --- | --- |
| Norway Spruce | *Picea abies* | 7,6 | Data from a project studying a north-south transect through Europe. Sites were located in Sweden (1 site), Denmark (2), Czech Republic (1), Germany (1), France (1) and Italy (1)  **Living biomass:** 20–30 sampled trees. Trees 31–180 years old, and from planted and natural stands.  **Soil:** Soil cores up to 20 cm, values exclude the litter layer. Excludes a site in Denmark | (Scarascia-Mugnozza *et al.*, 2000)  (Harrison *et al.*, 2000) |
| Scots Pine | *Pinus sylvestris* | 63 | **Living biomass:** 63 plots along a Mountain range in Turkey; Trees 8–177 years old.  **Soil:** A core was taken at each sampling plot. Soil depth up to 1 m. Values exclude the “litter layer” (Horizon O and aboveground dead wood). | (Lee *et al.*, 2016) |
| Beech | *Fagus sylvatica* | 5,4 | Data from a project studying a north-south transect through Europe. Sites were located in Denmark (1 site), Czech Republic (1), Germany (1), France (1) and Italy (1).  **Living biomass:** 20–30 sampled trees. Trees 79–161 years old, and from planted and natural stands.  **Soil:** Soil cores up to 20 cm, values exclude the litter layer. Excludes the site in the Czech Republic | (Scarascia-Mugnozza *et al.*, 2000)  (Harrison *et al.*, 2000) |
| Temperate oak | *Quercus robur* | 4 | **Living biomass:** Four mature even-aged stands of *Q. robur* in NW Spain. Low degree of silvicultural intervention  **Soil:** Three cores were taken at each of the described stands. Soil depths varied from 0–110 cm depth, and values exclude the “litter layer” (Horizon O). | (Balboa-murias *et al.*, 2006) |
| Mediterranean oak | *Quercus ilex* | 151,21 | **Living biomass:** 151 plots along a mountain range in NE Spain. The authors assessed the above-ground tree biomass (i.e. 104.2 t ha^-1^) and estimated the underground biomass using the findings of (Canadell & Rodà, 1989), considering it to be 85% of the aerial biomass (i.e. 95.5 t ha^-1^). Values were then converted to tC ha^-1^ using the mean carbon content of temperate broadleaf trees as per (Thomas & Martin, 2012).  **Soil:** 21 sites distributed across mainland Spain. *Q. ilex* formed “dense forests” and was the dominant species at all sites. Values are for the total soil carbon in the top soil (0–20 cm). | (Lledó *et al.*, 1992)  (González González *et al.*, 2012) |
| Tidal Marsh | *Elytrigia atherica* syn. *Elymus athericus* | 2 | **Living biomass:** Two sampling sites at different elevations from a brackish water tidal marsh in the Netherlands.  **Soil:** Three cores taken at each site; soil depths varied from 0.75–1.4m, data presented is profiles down to 0.6 m.  *Note: The study also investigated a saltwater and a freshwater marsh, which stored a mean total of 106.5 and 211.5 tC · ha^-1^ respectively | (Van de Broek *et al.*, 2016) |
| Seagrass | *Posidonia oceanica* | 57,29 | **Living biomass:** From a review of different studies performed in the Mediterranean Sea. Values correspond to the amount of organic carbon stored in living seagrass biomass.  **Soil:** From the same review. Values are for organic C down to 1 m depth. Some of the values may have been estimated (see reference for details). | (Fourqurean *et al.*, 2012)  (Fourqurean *et al.*, 2012) |
| Kelp | *Laminaria hyperborea* | 8 | **Living biomass:** 8 kelp forests along a latitudinal gradient in the UK (two forests per location).  **Soil:** No data available, thought to be negligible | This study |

**Table S10** Data sources used to quantify the carbon flux via detritus associated with dominant European habitat-forming species, as shown in Fig. 4.

| **Common name** | **Species** | **n** | **Study sites and descriptions** | **Reference** |
| --- | --- | --- | --- | --- |
| Norway Spruce | *Picea abies* | 44 | Data compiled from a review from 17 different studies of European temperate forests, comprising 1–61 sampled stands per study. Litterfall biomass was then converted to carbon content using the ratios for stem wood provided in (Thomas & Martin, 2012). This was 0.488 for Temperate/Boreal broadleaf trees, 0.508 for Temperate/Boreal coniferous trees | (Augusto *et al.*, 2002) |
| Scots Pine | *Pinus sylvestris* | 20 |  |  |
| Beech | *Fagus sylvatica* | 43 |  |  |
| Temperate oak | *Quercus robur* | 15 |  |  |
| Mediterranean oak | *Quercus ilex* | 1 | Data from a 7-year long study of a forest in Catalonia, NW Spain. Values were then converted to carbon using the mean carbon content of temperate broadleaf trees as per (Thomas & Martin, 2012). | (Bellot *et al.*, 1992) |
| Tidal marsh | *Elytrigia atherica* | 1 | Values for a high marsh in France. Detritus production was estimated as the product between net aerial primary production and turn over rate. | (Bouchard & Lefeuvre, 2000) |
| Seagrass | *Posidonia oceanica* | 3 | Value corresponds to the leaf production at three sampling sites (5, 10 and 20 m depth) along a meadow in Italy. The estimate assumes that leaf herbivory was minimal, and there was no loss of rhizomatous tissue. | (Pergent *et al.*, 1994) |
| Kelp | *Laminaria hyperborea* | 8 | 8 kelp forests along a latitudinal gradient in the UK (two forests per location). | This study |

**Table S11** Rates of detritus production associated with the May cast shedding of old lamina tissue (g C · m^-2^ · year^-1^). Real values of mean and S.D. were used in the Monte Carlo simulations, which produced 1000 randomly generated simulated values of May cast detritus production following a normal distribution. Those values were then used to estimate ‘chronic’ erosion.

| **Location** | **C1** | | **C2** | | **W1** | | **W2** | |
| --- | --- | --- | --- | --- | --- | --- | --- | --- |
| **Site** | **A** | **B** | **C** | **D** | **E** | **F** | **G** | **H** |
| REAL VALUES | | | | | | | | |
| Mean | 210.8 | 210.4 | 121.3 | 51.2 | 52.2 | 33.3 | 92.4 | 70.5 |
| SD | 86.1 | 86.1 | 51.8 | 26.9 | 27.2 | 15.6 | 80.0 | 33.9 |
| Min | 53.0 | 49.7 | 32.3 | 13.2 | 17.9 | 17.0 | 24.2 | 13.0 |
| Max | 326.6 | 337.7 | 241.9 | 105.8 | 98.5 | 59.6 | 241.8 | 149.4 |
| SE | 21.5 | 21.5 | 11.0 | 6.7 | 7.9 | 4.5 | 21.4 | 9.4 |
| SIMULATED VALUES | | | | | | | | |
| Mean | 212.2 | 213.0 | 121.3 | 53.1 | 50.9 | 33.3 | 85.9 | 70.7 |
| SD | 87.6 | 88.0 | 50.9 | 26.8 | 27.7 | 15.9 | 80.4 | 33.7 |
| Min | 38.7 | 12.3 | -45.4 | -3.6 | -6.2 | -20.7 | -186.8 | -3.1 |
| Max | 395.8 | 448.0 | 232.1 | 141.0 | 105.8 | 69.5 | 289.7 | 144.9 |

**Table S12** Rates of annual detritus production associated with ‘chronic’ erosion of the lamina tissue (g C · m^-2^ · year^-1^). Estimates were obtained by randomly assigning a percentage contribution (between 56-70%) to a randomly obtained May cast production value (see above).

| **Location** | **C1** | | **C2** | | **W1** | | **W2** | |
| --- | --- | --- | --- | --- | --- | --- | --- | --- |
| **Site** | **A** | **B** | **C** | **D** | **E** | **F** | **G** | **H** |
| Mean | 136.7 | 136.4 | 76.4 | 33.9 | 32.3 | 21.3 | 55.5 | 45.0 |
| SD | 62.8 | 64.9 | 37.2 | 18.7 | 19.3 | 11.3 | 54.2 | 23.6 |
| Min | 16.9 | 6.9 | -29.6 | -1.9 | -4.0 | -13.5 | -158.3 | -2.2 |
| Max | 320.4 | 279.0 | 190.6 | 113.2 | 84.0 | 54.1 | 201.1 | 125.0 |


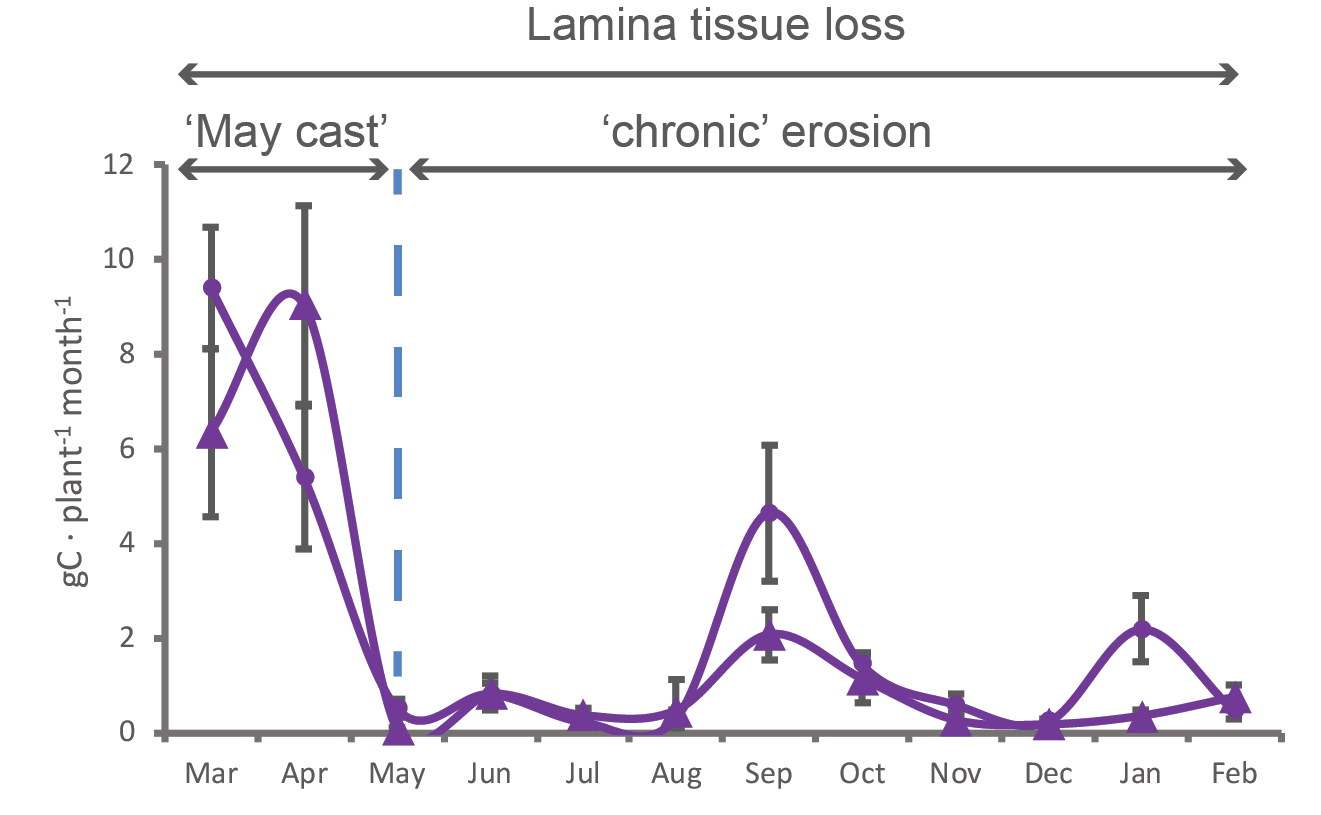


**Figure S1** Kelp plant lamina tissue loss throught the year. Loss occurs via two mechanisms, the shedding of the old lamina growth collar (‘May cast’), and gradual erosion of the lamina tips throught the rest of the year (‘chronic’ erosion). Data from monthly observations of two independent populations in southwest England.


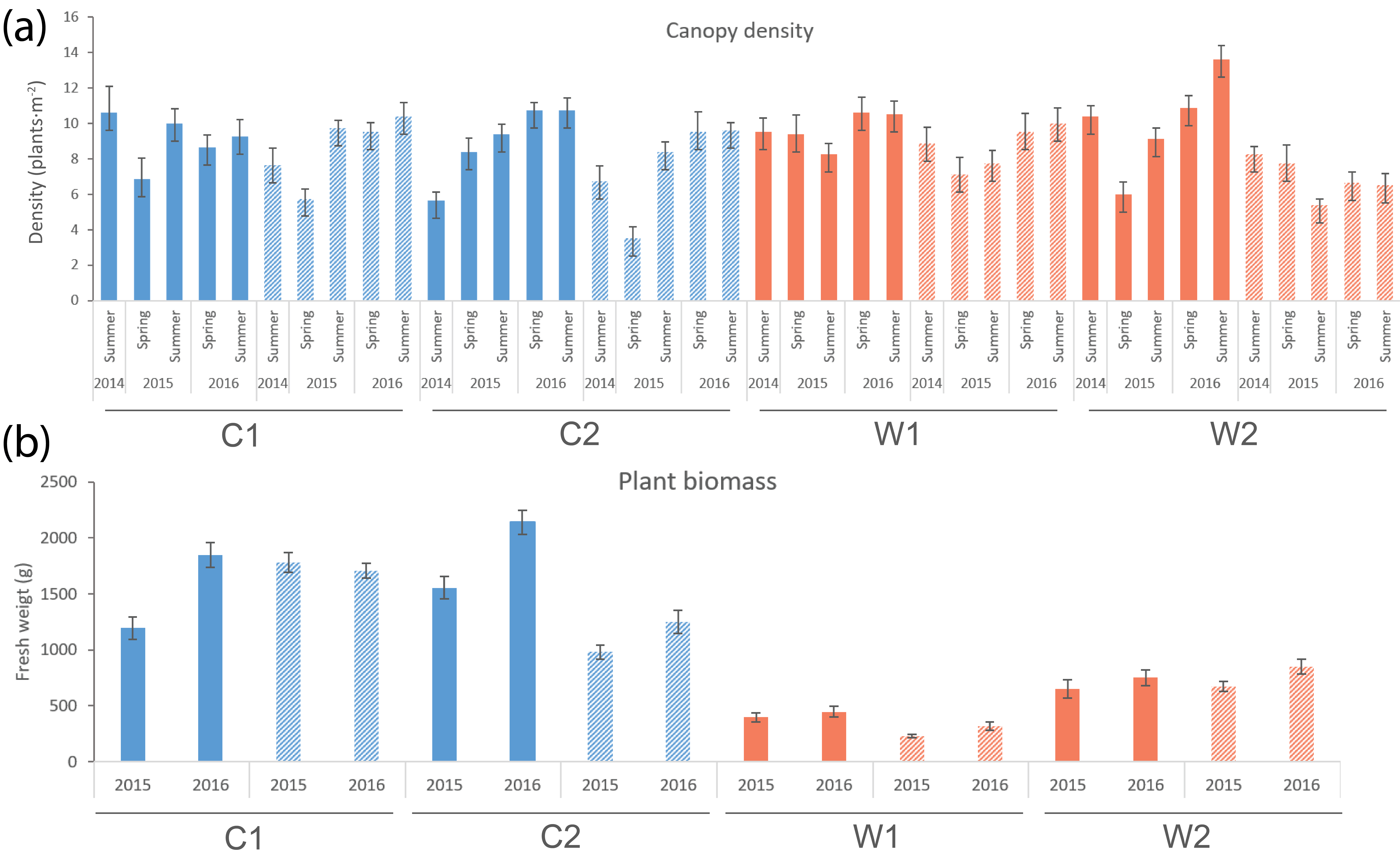


**Figure S2** Kelp plant density (a) and fresh weight biomass (b) at our cold (blue; C1,C2) and warm (orange; W1, W2) locations. Filled and striped bars indicate the two survey sites within each location. For the density surveys, 8 quadrats were placed in summer and spring during 2014–2016. In summer 2015 and 2016, 15 canopy-forming *Laminaria hyperborea* individuals were weighed.


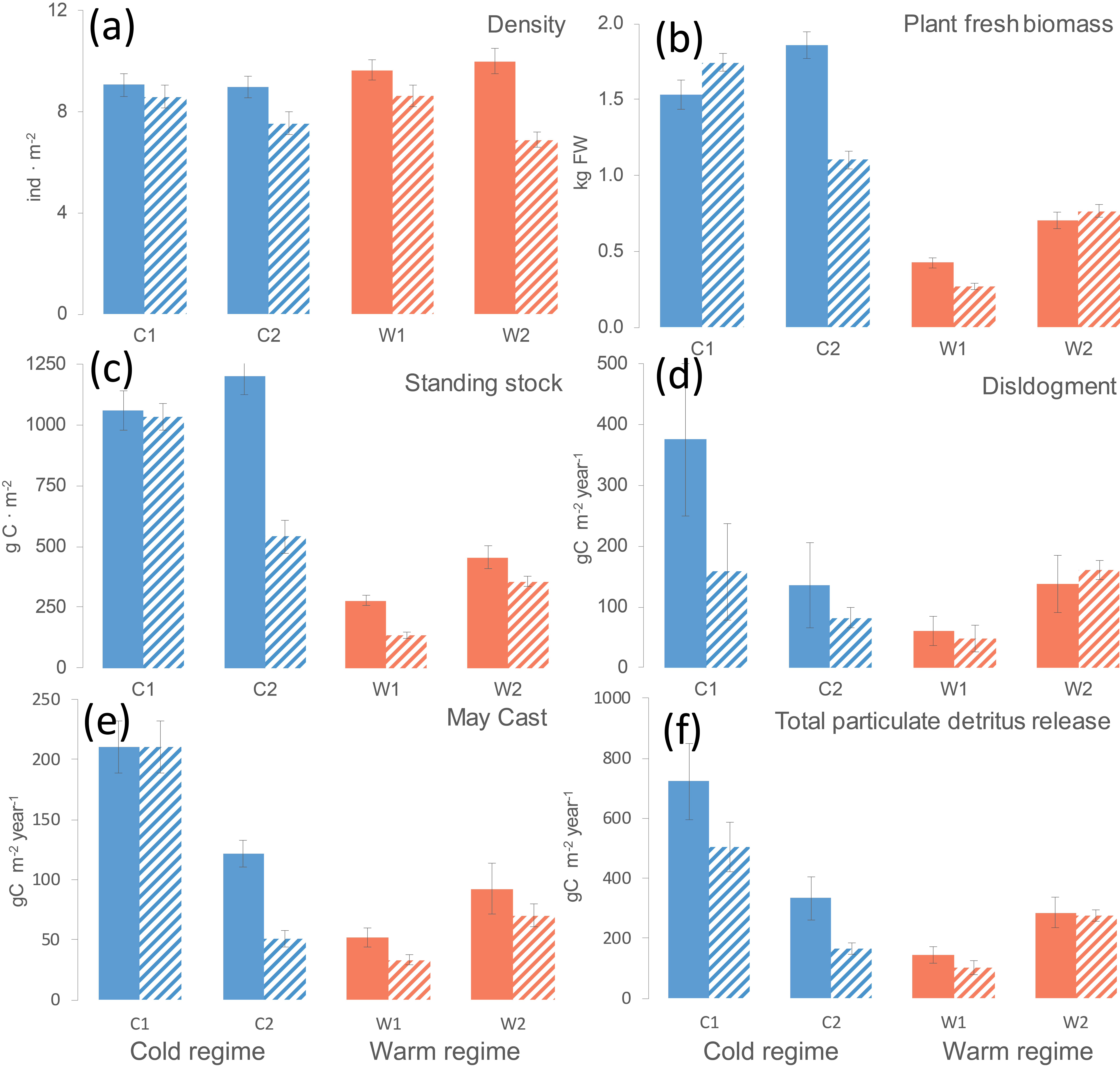


**Figure S3** Mean kelp density (a), individual kelp plant biomass (b), standing stock of carbon (c) and detrital production (d–f) at each the surveyed sites for years between 2014–2016. Bars represent average values ± standard error (SE) at the cold (blue; C1, C2) and warm (orange; W1, W2) locations. Filled and striped bars indicate the two survey sites within each location.


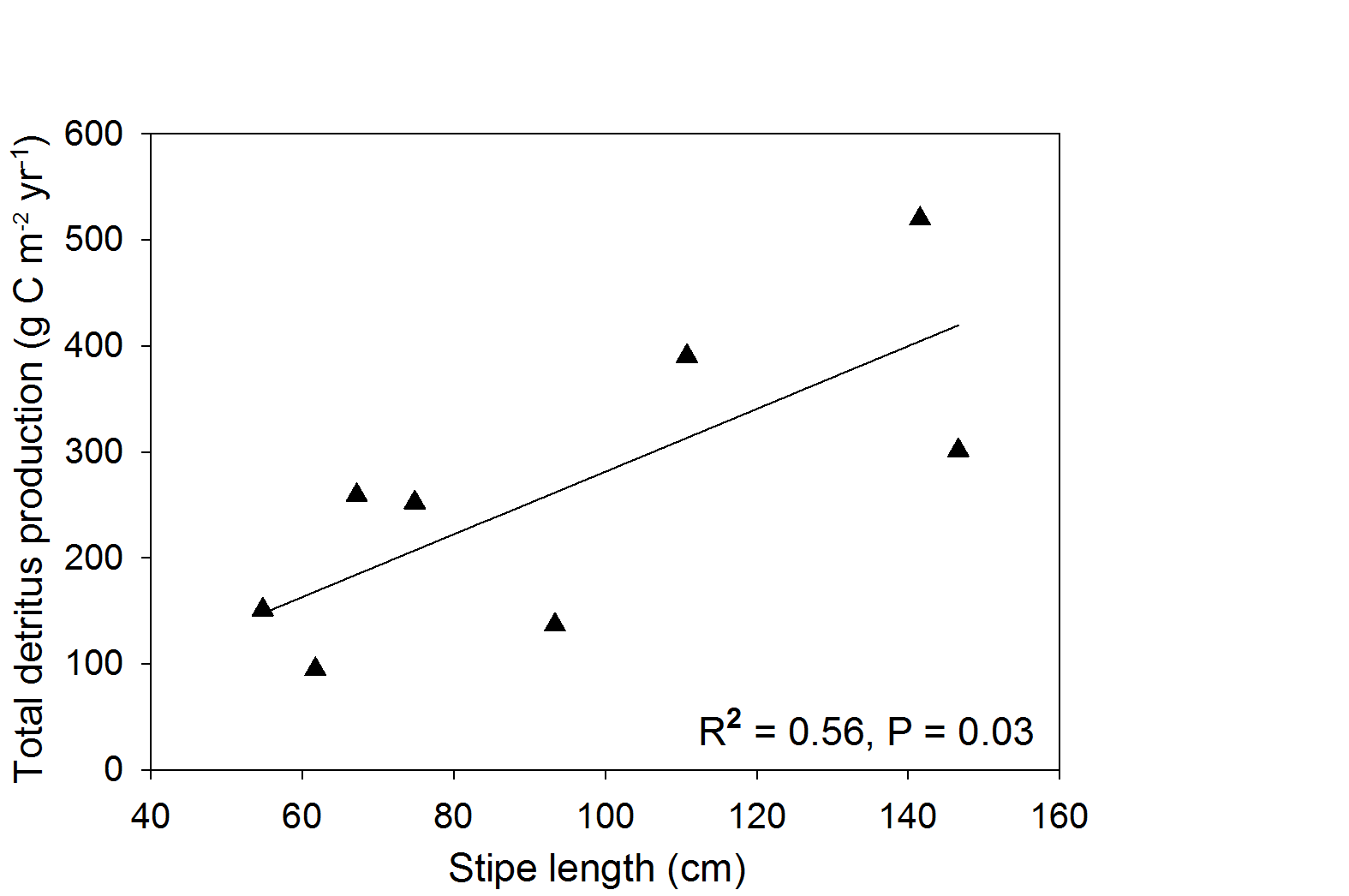


**Figure S4** The relationship between mean stipe length and total annual detritus production for the eight study sites in the UK.

**References**

Assis J, Lucas AV, Bárbara I, Serrão EA (2016) Future climate change is predicted to shift long-term persistence zones in the cold-temperate kelp Laminaria hyperborea. *Marine Environmental Research*, **113**, 174–182.

Augusto L, Ranger J, Binkley D, Rothe A (2002) Impact of several common tree species of European temperate forests on soil fertility. *Annals of Forest Science*, **59**, 233–253.

Balboa-murias MA, Rojo A, Alvarez JG et al. (2006) Carbon and nutrient stocks in mature Quercus robur L. stands in NW Spain. *Annals of Forest Science*, **63**, 577–565.

Bekkby T, Rinde E, Gundersen H, Norderhaug KM, Gitmark JK, Christie HC (2014) Length, strength and water flow: Relative importance of wave and current exposure on morphology in kelp Laminaria hyperborea. *Marine Ecology Progress Series*, **506**, 61–70.

Bellot J, Sánchez JR, Lledó MJ, Martínez P, Escarré A (1992) Litterfall as a measure of primary production in Mediterranean holm-oak forest. *Vegetatio*, **99**–**100**, 69–76.

Bouchard V, Lefeuvre J-C (2000) Primary production and macro-detritus dynamics in a European salt marsh: carbon and nitrogen budgets. *Aquatic Botany*, **67**, 23–42.

Van de Broek M, Temmerman S, Merckx R, Govers G (2016) Controls on soil organic carbon stocks in tidal marshes along an estuarine salinity gradient. *Biogeosciences*, **13**, 6611–6624.

Burrows MT (2012) Influences of wave fetch, tidal flow and ocean colour on subtidal rocky communities. *Marine Ecology Progress Series*, **445**, 193–207.

Burrows MT, Kamenos NA, Hughes DJ, Stahl H, Howe JA, Tett P (2014) *Assessment of carbon budgets and potential blue carbon stores in Scotland’s coastal and marine environment*.

Canadell J, Rodà F (1989) Biomasa y mineralomasa subterránea del encinar de La Castanya, Montseny. *Options Méditerranéennes Serie A*, **3**, 13–18.

Chassé C, Kerambrun L (1988) Le champ d’algues Breton et son potentiel économique: répartition, espèces, biomasses et production. In: *Valorisation des algues et autres végétaux aquatiques* (eds Delépine R, Gaillard R, Morand P), pp. 119–122. IFREMER, Brest.

Dye SR, Holliday NP, Hughes SL et al. (2013) Climate change impacts on the waters around the UK and Ireland: Salinity. *MCCIP Science Review*, 60–66.

Fourqurean JW, Duarte CM, Kennedy H et al. (2012) Seagrass ecosystems as a globally significant carbon stock. *Nature Geoscience*, **5**, 505–509.

González González I, Grau Corbí JM, Fernández Cancio A, Jiménez Ballesta R, González Cascón MR (2012) Soil carbon stocks and soil solution chemistry in Quercus ilex stands in Mainland Spain. *European Journal of Forest Research*, **131**, 1653–1667.

Gundersen H, Christie H, de Wit H, Norderhaug K, Bekkby T, Walday M (2010) *Utredning om CO2-opptak i marine naturtyper*. 27 pp.

Gunnarsson K (1991) Population de Laminaria hyperborea et Laminaria digitata dans le Baie de Breidafjördur, Island. *Journal of the Marine Research Institute Reykjavik*, **12**, 113.

Harrison AF, Harkness DD, Rowland AP, Garnett JS, Bacon PJ (2000) Annual Carbon and Nitrogen Fluxes in Soils Along the European Forest Transect, Determined Using 14C-Bomb. In: *Carbon and nitrogen cycling in European forest ecosystems* (ed Schulze E-D), pp. 237–256. Springer Berlin Heidelberg, Berlin.

Jensen A (1998) The seaweed resources of Norway. In: *Seaweed Resources of the World* (eds Critchley AT, Ohno M), pp. 200–209. Japan International Cooperation Agency, Yokosuka.

John DM (1968) *Studies on littoral and sublittoral ecosystems*. 168 pp.

Jupp BP, Drew EA (1974) Studies on the growth of Laminaria hyperborea (Gunn.) Fosl. I. Biomass and productivity. *Journal of Experimental Marine Biology and Ecology*, **15**, 185–196.

Kain JM (1963) Aspects of the biology of Laminaria hyperborea II. Age, weight and length. *Journal of the Marine Biological Association of the UK*, **43**, 129–151.

Kain JM (1977) The biology of *Laminaria hyperborea* X. The effect of depth on some populations. *Journal of the Marine Biological Association of the United Kingdom*, **57**, 587–607.

Lee J, Makineci E, Son YM (2016) Estimating the age-dependent changes in carbon stocks of Scots pine ( Pinus sylvestris L .) stands in Turkey. *Annals of Forest Science*.

Lledó JM, Sánchez JR, Bellot J, Boronat J, Ibañez JJ, Escarré A (1992) Structure, biomass and production of a resprouted holm-oak (Quercus-ilex L.) forest in NE Spain. *Vegetatio*, **100**, 51–59.

Norton TA, Hiscock K, Kitching JA (1977) The Ecology of Lough Ine: XX. The Laminaria Forest at Carrigathorna. *Journal of Ecology*, **65**, 919–941.

Pedersen MF, Nejrup LB, Fredriksen S, Christie HC, Norderhaug KM (2012) Effects of wave exposure on population structure, demography, biomass and productivity of the kelp Laminaria hyperborea. *Marine Ecology Progress Series*, **451**, 45–60.

Pergent G, Romero J, Pergentmartini C, Mateo MA, Boudouresque CF (1994) Primary production, stocks and fluxes in the Mediterranean seagrass Posidonia oceanica. *Marine Ecology Progress Series*, **106**, 139–146.

Rinde E, Sjøtun K (2005) Demographic variation in the kelp Laminaria hyperborea along a latitudinal gradient. *Marine Biology*, **146**, 1051–1062.

Scarascia-Mugnozza G, Bauer GA, Persson H, Matteucci G, Masci A (2000) Tree Biomass, Growth and Nutrient Pools. In: *Carbon and nitrogen cycling in European forest ecosystems* (ed Schulze E-D), pp. 49–62. Springer Berlin Heidelberg, Berlin.

Sheppard CRC, Jupp BP, Sheppard ALS, Bellamy DJ (1978) Studies on the growth of Laminaria hyperborea (Gunn.) Fosl. and Laminaria ochroleuca De la Pylaie on the French channel coast. *Botanica Marina*, **21**, 109–116.

Sjøtun K, Fredriksen S, Lein TE, Rueness J, Sivertsen K (1993) Population studies of Laminaria hyperborea from its northern range of distribution in Norway. *Hydrobiologia*, **260**–**261**, 215–221.

Sjøtun K, Christie H, Fosså JH (2000) Ressursgrunnlaget for taretråling og gjenvekst etter prøvetråling i Sør-Trøndelag. *Fisken Havet*, **6**, 1–27.

Smale DA, Burrows MT, Evans AJ, King N, Sayer MDJ, Yunnie ALE, Moore PJ (2016) Linking environmental variables with regional-scale variability in ecological structure and standing stock of carbon within kelp forests in the United Kingdom. *Marine Ecology Progress Series*, **542**, 79–95.

Thomas SC, Martin AR (2012) Carbon content of tree tissues: A synthesis. *Forests*, **3**, 332–352.

Werner A, Kraan S (2004) *Review of the potential mechanisation of kelp harvesting in Ireland*. 52 pp.

Whittock A (1969) *The kelp forest ecosytem at Petticoe Wick Bay lat 55°55’N. long 2°09’W: an ecological study*. University of Durham, 139 pp.

Yesson C, Bush LE, Davies AJ, Maggs C, Brodie J (2014) The distribution and environmental requirements of large brown seaweeds in the British Isles. *Journal of the Marine Biological Association of the UK*, 1–12.
